# Influence of psoas major morphology on clinical outcomes following L4-5 oblique lumbar interbody fusion: a retrospective study of 191 patients

**DOI:** 10.3389/fmed.2026.1860872

**Published:** 2026-08-19

**Authors:** Xinpeng Zhou, Lianghai Jiang, Yunfan Lyu, Lantao Liu

**Affiliations:** 1 Department of Spine Surgery, Qingdao Municipal Hospital, Qingdao University, Qingdao, Shandong, China; 2 Department of Spine Surgery, Qingdao Municipal Hospital, Qingdao, Shandong, China; 3 Department of Joint Surgery, Qingdao Municipal Hospital, Qingdao University, Qingdao, Shandong, China

**Keywords:** clinical efficacy, interior elevation, oblique lumbar interbody fusion, operative window, psoas major morphology

## Abstract

**Background:**

Oblique lumbar interbody fusion (OLIF) has emerged as a widely adopted minimally invasive surgical technique in recent years. However, the relationship between the positional characteristics and morphological configuration of the psoas major (PM) relative to the vertebral body and the specific influence of psoas major morphology on surgical outcomes remains incompletely characterized. This study aims to investigate the impact of the elevated and apposed PM on the efficacy of OLIF.

**Methods:**

A retrospective analysis was conducted on 191 consecutive patients who underwent L4-5 OLIF between January 2022 and January 2025. All the patients had a minimum follow-up duration of 12 months. A novel parameter, the interior elevation (IE), was introduced. Based on the IE index, patients were divided into two groups: the elevated type and the apposed type. The relevant key parameters in accordance with the expressions below: The operative window (OW), psoas major sagittal diameter (PMSD), major transverse diameter (PMTD), psoas major cross-sectional area (PMCSA), psoas major hematoma area (PMHA), central spinal canal area (CSCA) were compared. Binary logistic regression analysis was conducted to identify imaging-related predictors of clinical outcomes following OLIF.

**Result:**

This study enrolled a total of 191 patients, all of whom were followed up for a 1-year period. Patients with the apposed-type PM demonstrated a larger post-operative PMCSA (1448.52 ± 269.97 vs. 1242.42 ± 331.01, *p* = 0.007), a higher degree of PM swelling percentage (38.42 ± 7.11% vs. 14.38 ± 4.70%, *p* = 0.005) and a higher incidence of transient post-operative complications (20.80% vs. 9.50%, *p* = 0.012). Moreover, these patients exhibited higher VAS scores at 1 week post-operatively (VASL: 4.71 ± 0.81 vs. 3.82 ± 0.75, *p* < 0.001), (VASB: 4.32 ± 0.47 vs. 3.67 ± 0.80, *p* = 0.024). In the Univariate regression analysis of multiple variables, the incidence of transient post-operative complications was significantly associated with OW (odds ratio, 0.641; *p* = 0.004) and IE (odds ratio, 0.882; *p* < 0.001).

**Conclusion:**

We propose a novel classification method for the psoas major muscle based on IE. The apposed-type PM, characterized by a smaller IE and a narrower OW, is associated with a higher incidence of post-operative complications.

## 1 Introduction

Oblique lumbar interbody fusion (OLIF) has emerged as a widely adopted minimally invasive surgical technique in recent years. This approach accesses the intervertebral disc space through the natural anatomical corridor between the anterior border of the psoas major (PM) and the abdominal vessels within the retroperitoneal space, allowing for disc removal and interbody fusion (1–3). Although the technique was first described by Mayer (4) in 1997, it did not gain widespread adoption at that time. In 2012, Silvestre et al. (5) formally introduced the term “OLIF” and reported its clinical outcomes. By enabling the placement of a larger interbody cage, OLIF restores disc height while maintaining spinal stability, thereby achieving indirect decompression and segmental fusion (6, 7). Furthermore, this approach preserves posterior spinal structures, including the facet joints, spinous processes, interspinous ligaments, and posterior longitudinal ligament, while avoiding dissection of the paraspinal musculature and incision of the PM. These advantages contribute to a reduced incidence of muscle-related post-operative complications (1, 2, 8). Currently, the indications for OLIF continue to expand. The cases previously considered unsuitable for OLIF (e.g., severe lumbar spinal stenosis) may now be managed with OLIF in combination with modified surgical techniques (9).

Despite these advantages, OLIF remains associated with a risk of neural and muscular injury, with reported perioperative and post-operative complication rates ranging from 11.7% to 36.4% (3, 10). Common complications include hip flexion weakness, anterior thigh sensory disturbances, sympathetic chain injury, and psoas hematoma formation. Among these, psoas-related complications are most frequently manifested as post-operative hip flexion weakness (11). Previous studies have suggested that these complications are closely associated with the degree of PM injury and hematoma formation (12). Factors contributing to psoas injury include difficulty in psoas mobilization, psoas hypertrophy, and narrowing of the operative corridor (13). These issues are particularly pronounced at the L4-5 level, where the PM is typically bulkier and more anteriorly positioned (12, 14). Therefore, the morphology of the PM, as well as its spatial relationship with the vertebral body, may play a critical role in determining clinical outcomes and complication profiles. Moro et al. (15) proposed the Moro zones based on the cross-sectional morphology of the psoas major muscle. Based on the method, the axial view of the intervertebral disc was divided into six regions, and the location of the left PM was evaluated. Zones I to IV were of equal width. Beyond the anterior and posterior edge of the disc, the two regions were defined as Zone A and Zone P, respectively. Louie et al. (16) verified the incidence of teardrop-shaped psoas major using a large imaging sample. However, the impact of psoas morphology on OLIF outcomes has rarely been investigated. Elucidating the influence of psoas configuration, particularly its appositional relationship with the vertebral body, may provide important insights for reducing psoas-related complications during and after surgery.

Accordingly, in the present study, we introduce a novel parameter, the interior elevation (IE), to classify the PM at the L4-5 level into elevated and apposed types. We aim to investigate the impact of these two psoas configurations on the efficacy of OLIF.

## 2 Materials and methods

### 2.1 Ethical statement

This study was approved by the Ethics Committee of Qingdao Municipal Hospital (Approval No. 2025-KY-059-062) and was conducted in accordance with the *Declaration of Helsinki*. Before study initiation, all participants were informed of the study objectives, scope, and intended use of data. All data were handled confidentially and used exclusively for research purposes. Participation was voluntary, and all patients were informed of their right to withdraw from the study at any time. Written informed consent was obtained from all participants before enrollment.

### 2.2 Patients

**Figure 1:**
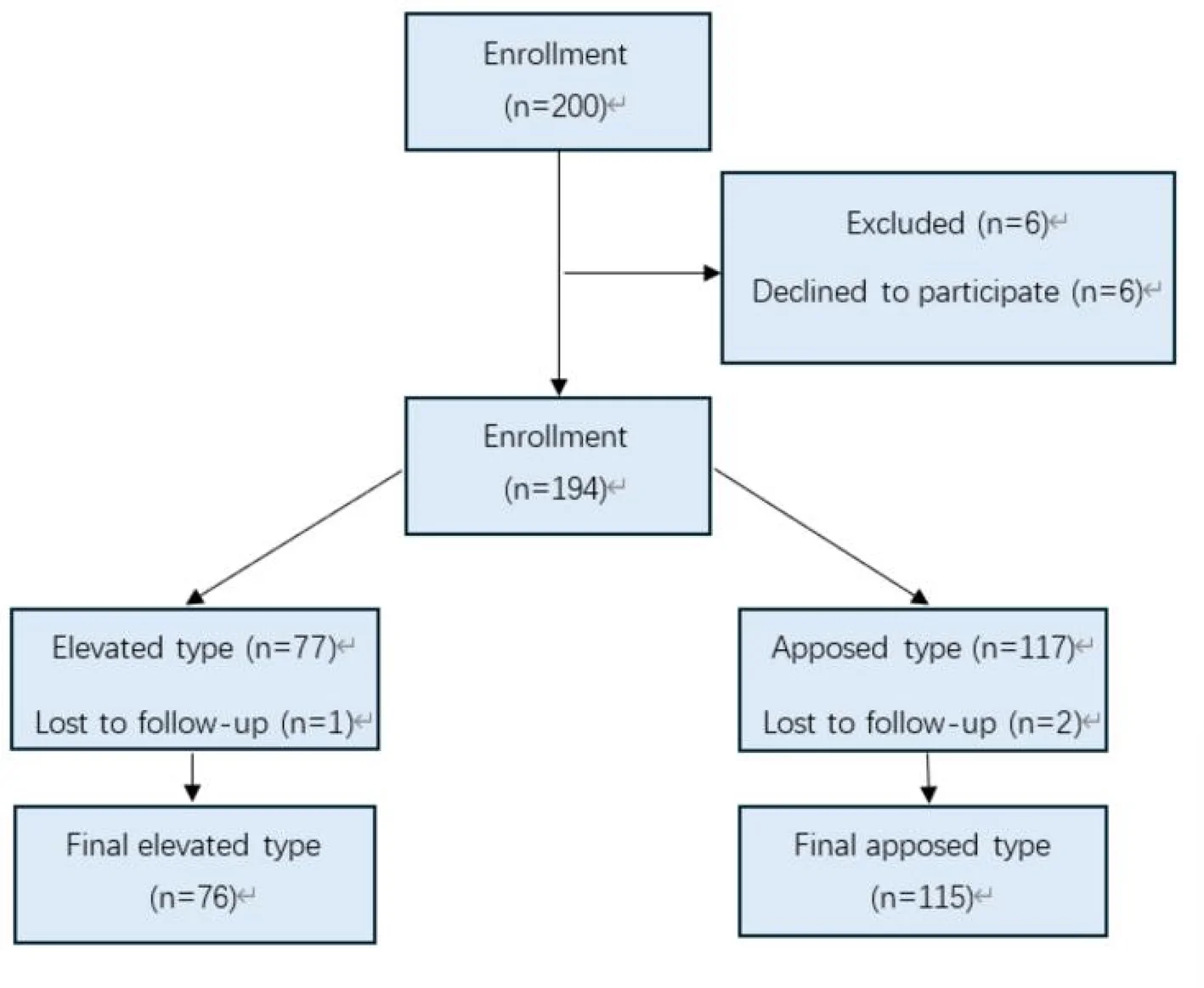
Flow chart for patient grouping.

A retrospective analysis was performed on 200 consecutive patients with degenerative lumbar spine disorders who underwent L4-5 OLIF between January 2022 and January 2025. Inclusion criteria were as follows: age between 18 and 80 years; Grade I or II lumbar spondylolisthesis; lumbar instability; lumbar spinal stenosis; or L4-5 discogenic low back pain with intermittent claudication (symptom relief >70% after rest); failure of conservative treatment for more than 3 months; and a minimum follow-up duration of 12 months. Exclusion criteria included: scoliosis; massive lumbar disc herniation or sequestration; severe lumbar spinal stenosis; spinal tumors or fractures; prior lumbar surgery at the same level; severe comorbidities or advanced osteoporosis; a history of abdominal or retroperitoneal surgery; and loss to follow-up. Six patients declined participation, and 3 patients were lost to follow-up. A total of 191 patients were finally included in the statistical analysis. The same spine surgery team performed all surgical procedures (Figure 1).

### 2.3 Intervention

**Figure 2:**
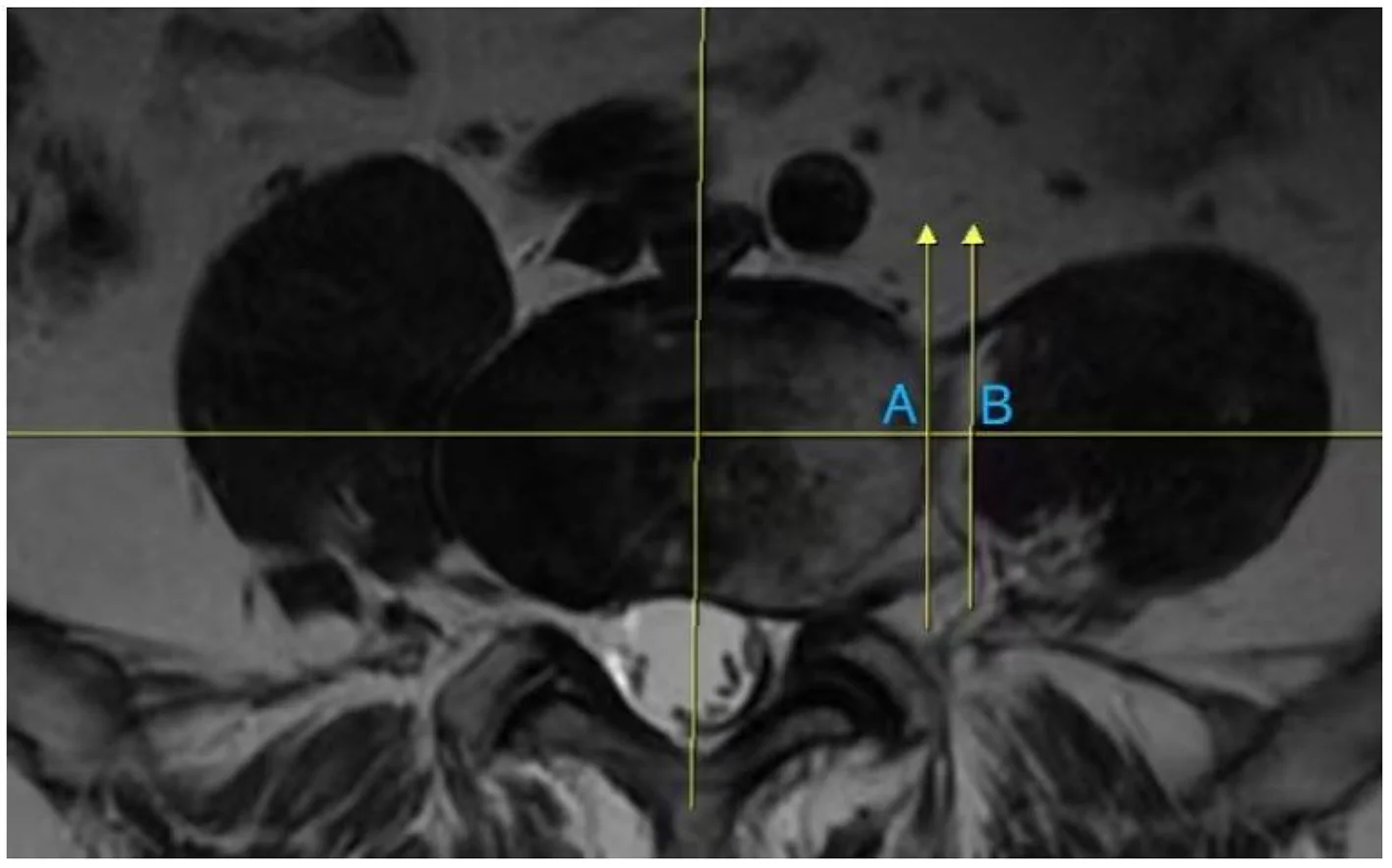
Classification criteria. Interior elevation **(A, B)**.

Based on the appositional relationship between the PM and the vertebral body along the left transverse axis at the midpoint of the L4-5 intervertebral disc, the IE was defined as the perpendicular distance from the medial border of the PM to the point of intersection between the left transverse axis of the L4-5 disc and its lateral margin. Berry (17) reported that the mean effective retroperitoneal surgical corridor was merely 6.1 mm in patients with IE ≤ 3 mm, and the intraoperative traction duration of the psoas major muscle was twice as long. Intraoperative exploration revealed no dissectible space between the psoas major and vertebral body when IE ≤ 3 mm (18, 19). Thus, the PM was classified into two types based on this parameter: an elevated type (IE > 3 mm) and an apposed type (IE ≤ 3 mm) (Figure 2).

### 2.4 Surgical technique

All patients were placed in the right lateral decubitus position under general anesthesia. Axillary and iliac padding was applied, allowing the abdomen to hang freely. After temporary spinal stabilization, the target level was confirmed using fluoroscopy. A standard oblique lateral approach was performed. The skin and subcutaneous tissues were incised sequentially, and the external oblique, internal oblique, and transversus abdominis muscles were bluntly dissected. The abdominal viscera and main vessels were mobilized anteriorly, while the PM was retracted posteriorly. The psoas major muscle was retracted by surgical assistants using standardized manual traction, without a standardized retraction system. Posterior fixation was performed during the same surgical session. The intervertebral disc space was then exposed, and the venous plexus on the disc surface was coagulated. A Kirschner wire was inserted, and fluoroscopy was used to reconfirm the target level. The left annulus fibrosus was incised, followed by the removal of the nucleus pulposus and cartilaginous endplates. Trial spacers were used to determine the optimal cage size. The selected cage, filled with allograft bone, was inserted under direct visualization, and its position was confirmed fluoroscopically. Finally, the muscle, subcutaneous tissue, and skin were closed in layers. The patient was subsequently repositioned to the prone position for posterior fixation.

### 2.5 Radiographic evaluation

**Figure 3:**
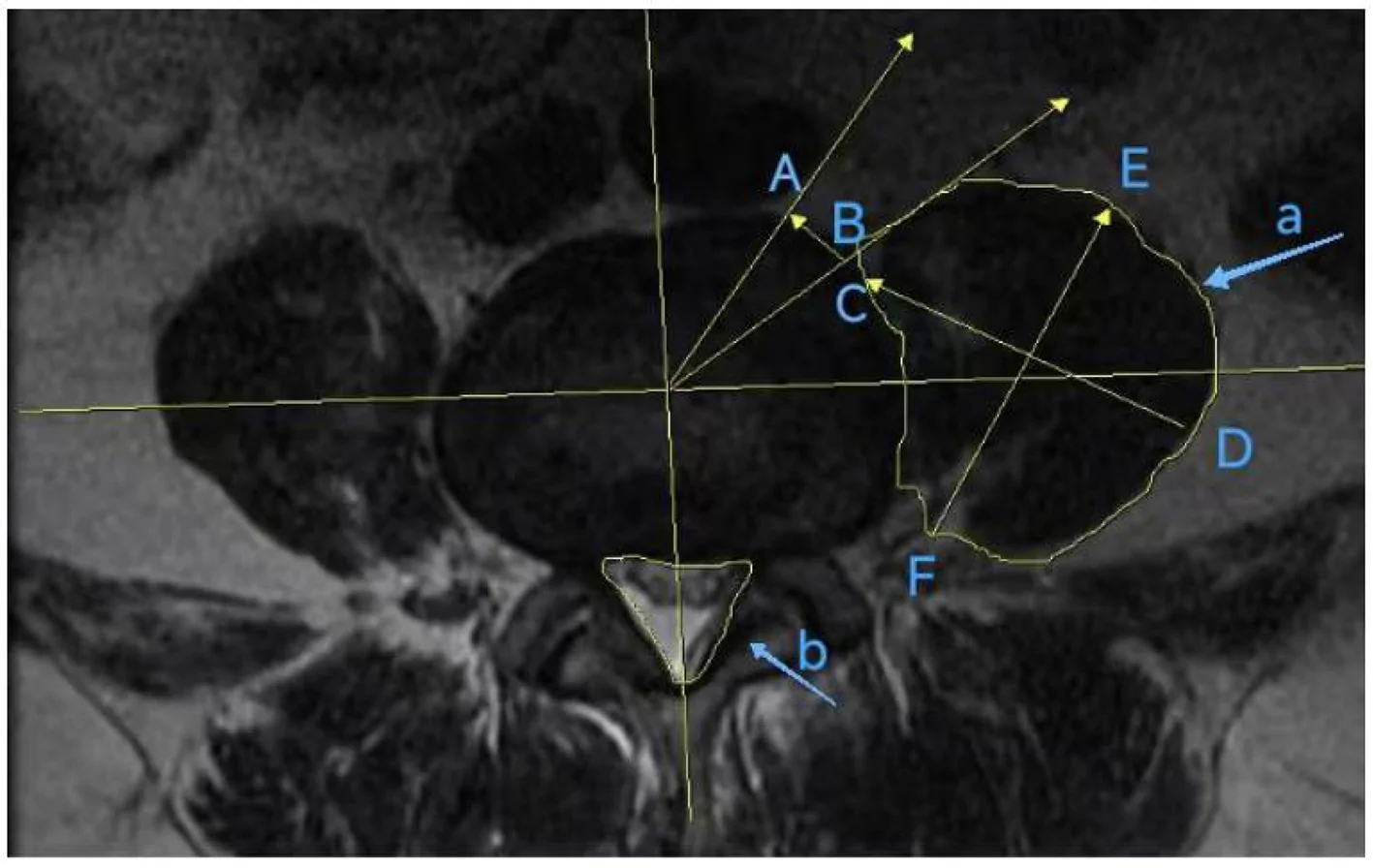
Cross-sectional parameters. operative window **(A, B)**, psoas major transverse diameter **(C, D)**, psoas major sagittal diameter **(E, F)**, psoas major cross-sectional area **(a)**, central spinal canal area **(b)**.

**Figure 4:**
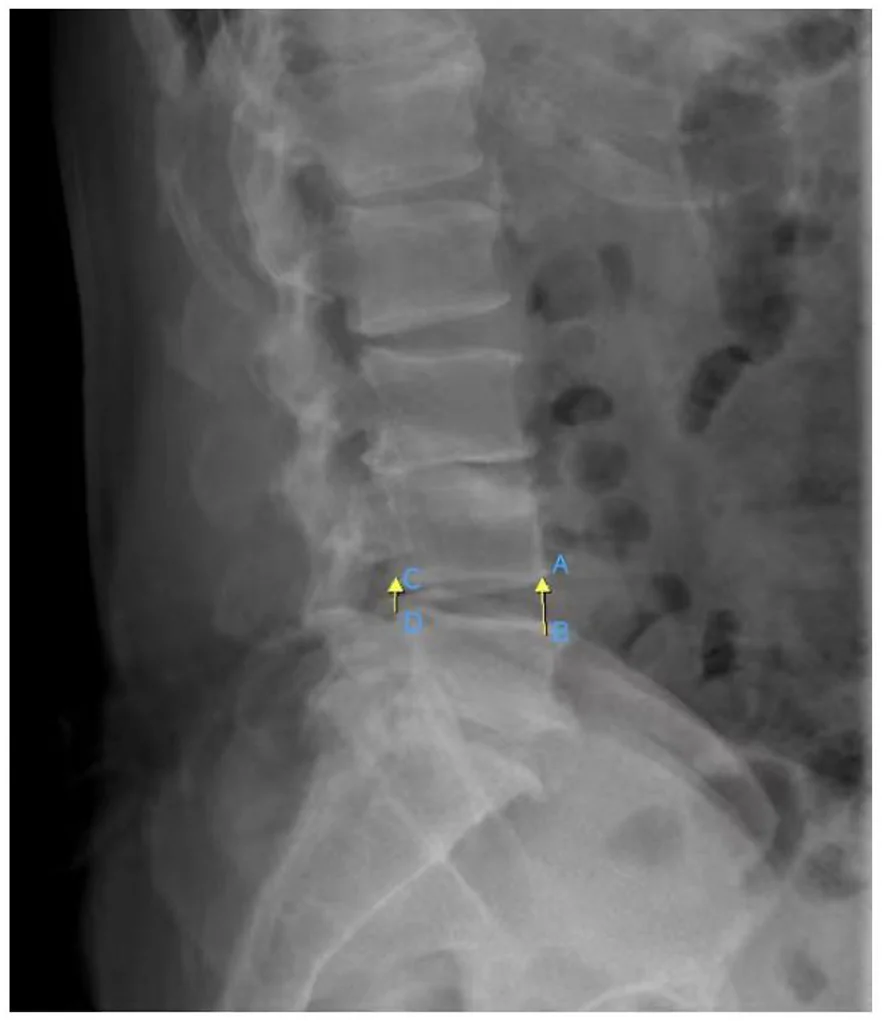
Sagittal parameters. Anterior disc height **(A, B)**, posterior disc height **(C, D)**.

Pre-operative and follow-up radiographs and magnetic resonance imaging (MRI) scans were obtained with patients in the supine position and analyzed using the Picture Archiving and Communication System (PACS). The following parameters were measured: operative window (OW), psoas major sagittal diameter (PMSD), psoas major transverse diameter (PMTD), psoas major cross-sectional area (PMCSA), psoas major hematoma area (PMHA), psoas swelling percentage, and central spinal canal area (CSCA) (Figure 3). Lateral radiographs were used to measure anterior disc height (ADH), posterior disc height (PDH), and the L4-5 segmental lordotic angle (SLA) (Figure 4). All radiographic measurements were performed in accordance with the method described by Siu et al. (20). The OW was defined as the anatomical space between the abdominal aorta and the psoas major muscle at the L4-5 segment. The PMSD is defined as the length of the psoas major muscle at the L4-5 cross-sectional level. The PMTD is defined as the width of the psoas major muscle at the L4-5 cross-sectional level. The psoas swelling percentage was defined as the ratio of the post-operative hematoma area of the psoas major muscle to the total cross-sectional area of the psoas major muscle. ADH is defined as the anterior intervertebral height, and PDH is defined as the posterior intervertebral height. SLA was calculated as the angle between tangent lines to the inferior endplate of the vertebra below the fused segment and the superior endplate of the vertebra above the fused segment. CSCA was defined as the area of the transverse dura mater capsule at the mid-level of the disc measured by axial T2-weighted MRI. Cage subsidence was defined as ≥2 mm vertical penetration of the cage into the superior or inferior vertebral endplate, compared with immediate post-operative baseline imaging. Cage migration refers to horizontal translational displacement of the interbody cage within the disc space relative to its immediate post-operative position, without vertical subsidence into vertebral endplates.

These parameters were measured independently by two spine surgeons. If discrepancies exist, measurements shall be performed by a senior spine surgeon. The intraclass correlation coefficient (ICC) was calculated to assess inter-rater agreement between the two spine surgeons. All ICC > 0.7. Radiographic evaluations were conducted within 1 month pre-operatively and at 3 days post-operatively.

### 2.6 Clinical outcome measures

Perioperative variables, including complications, estimated blood loss, operative time, and length of hospital stay, were recorded. Clinical outcomes were assessed using the Visual Analog Scale (VAS) and the Oswestry Disability Index (ODI) at 1 week, 1 month, 3 months, 6 months, and 12 months post-operatively.

### 2.7 Statistical analysis

Statistical analyses were performed using SPSS version 27.0 (SPSS Inc., Chicago, IL, USA). Data were screened for abnormalities and normalities using the Shapiro–Wilk test. Continuous variables were analyzed using the independent-samples *t*-test. Paired *t* tests were used to compare pre-operative and post-operative clinical and radiographic outcomes. Categorical variables were compared using the chi-square test or Fisher's exact test. Binary logistic regression analysis was performed to identify imaging-related predictors of clinical outcomes following OLIF. A *p*-value < 0.05 was considered statistically significant.

## 3 Results

### 3.1 Patient selection

A total of 200 patients initially met the inclusion criteria, of whom 9 were lost to follow-up. Ultimately, 191 patients were included in the final analysis. Among them, 76 patients were classified as the elevated-type group, including 31 males and 45 females, with a mean age of 62.33 ± 12.22 years. The apposed-type group comprised 115 patients, including 59 males and 56 females, with a mean age of 60.70 ± 8.51 years. No significant differences were observed between the two groups regarding sex, age, body mass index (BMI), bone mineral density (BMD), comorbidities, personal history, duration of symptoms, or primary diagnosis (Table 1).

**Table 1:** Summary of patient demographics.

Parameter	Elevated-type	Apposed-type	*p*-value
No. of patients	76	115	
No. of M/F	31/45	59/56	0.517
Age (years)	62.33 ± 12.22	60.70 ± 8.51	0.479
BMD	−2.25 ± 0.94	−2.07 ± 0.68	0.697
BMI(kg/m^2^)	24.12 ± 2.30	23.71 ± 2.51	0.587
Background diseases			
CVD	28(36.84%)	39(33.91%)	0.240
Diabetes	11(14.47%)	16(13.91%)	0.183
CVD and Diabetes	6(7.90%)	10(8.70%)	0.671
Long-term smoking	9(11.82%)	5(4.35%)	0.627
Main diagnosis			0.722
Spondylolisthesis	27	39	
Lumbar spinal stenosis	40	65	
Discogenic pain	9	11	

BMI, body mass index; BMD, bone mineral density; CVD, cardiovascular disease.

### 3.2 Perioperative outcomes

The operative time was 152.36 ± 32.54 min in the elevated-type group and 162.78 ± 22.57 min in the apposed-type group, with no statistically significant difference between the groups. Estimated blood loss was 65.30 ± 22.58 mL in the elevated-type group and 72.97 ± 24.89 mL in the apposed-type group, showing a statistically significant difference (*p* = 0.017). The length of hospital stay was 9.74 ± 2.98 days in the elevated-type group and 8.32 ± 1.93 days in the apposed-type group. The apposed-type group had a shorter hospital stay, and the difference was statistically significant (*p* = 0.028) (Table 2).

**Table 2:** Perioperative results of the elevated-type and the apposed-type.

Parameter	Elevated-type	Apposed-type	*p*-value
Operation time (minutes)	152.36 ± 32.54	162.78 ± 22.57	0.428
Estimated blood loss (ml)	65.30 ± 22.58	72.97 ± 24.89^*^	**0.017**
Length of hospital stay (days)	9.74 ± 2.98	8.32 ± 1.93^*^	**0.028**
Cost (RMB)	33572.41 ± 2882.68	32458.52 ± 3358.64	0.157
Complications			
Total complication rate	7(9.50%)	24(20.80%) ^*^	**0.012**
Cage displacement	0	1	0.682
Cage subsidence/endplate injury	0	0	–
Thigh pain/numbness, hip flexion weakness	6	23^*^	**0.015**
Sympathetic chain injury	1	0	0.427

^*^p < 0.05, compared with the elevated-type group. *p* < 0.05, statistically significant difference and *p* < 0.01, highly statistically significant difference indicates in bold.

### 3.3 Clinical outcomes

All patients underwent evaluation of subjective clinical outcomes pre-operatively and at 1 week, 1 month, 3 months, 6 months, and 12 months post-operatively. Significant improvements in VAS and ODI scores were observed during follow-up. Notably, at 1 week post-operatively, the Visual Analog Scale for back pain (VASB) and leg pain (VASL) scores were significantly lower in the elevated-type group compared with the apposed-type group (VASL: 3.82 ± 0.75 vs. 4.71 ± 0.81, *p* < 0.001), (VASB: 3.67 ± 0.80 vs. 4.32 ± 0.47, *p* = 0.024). The ODI scores were also lower in the elevated-type group compared with the apposed-type group (39.58 ± 3.87 vs. 41.48 ± 4.11, *p* = 0.031) (Table 3).

**Table 3:** Clinical comparison of the elevated-type and the apposed-type.

Parameter	Elevated-type	Apposed-type	*p*-value
VASL			
Pre-op	6.24 ± 1.00	6.83 ± 1.24	0.351
1-week post-op	3.82 ± 0.75	4.71 ± 0.81^₣^	**< 0.001**
1-months post-op	3.64 ± 0.62	3.27 ± 0.47	0.684
3-months post-op	3.19 ± 0.68	3.21 ± 0.42	0.544
6-months post-op	2.63 ± 0.51	2.46 ± 0.51	0.272
12-months post-op	2.14 ± 0.44	2.29 ± 0.46	0.315
VASB			
Pre-op	6.92 ± 1.21	7.43 ± 1.17	0.118
1-week post-op	3.67 ± 0.80	4.32 ± 0.47^*^	**0.024**
1-months post-op	3.01 ± 0.61	2.74 ± 0.57	0.142
3-months post-op	2.59 ± 0.72	2.33 ± 0.48	0.841
6-months post-op	2.12 ± 0.51	2.04 ± 0.51	0.564
12-months post-op	2.33 ± 0.48	2.42 ± 0.50	0.770
ODI (×100%)			
Pre-op	64.33 ± 7.88	63.88 ± 3.71	0.618
1-week post-op	39.58 ± 3.87	41.48 ± 4.11^*^	**0.031**
1-months post-op	33.51 ± 4.78	34.71 ± 3.71	0.544
3-months post-op	31.67 ± 4.29	32.77 ± 3.06	0.975
6-months post-op	20.10 ± 1.81	29.45 ± 1.61	0.364
12-months post-op	21.05 ± 0.87	21.36 ± 1.03	0.450

Pre-op, pre-operative; post-op: post-operative; VASB, visual analog scale of back; VASL, visual analog scale of leg; ^₣^*p* < 0.01, compared with the elevated-type group. ^*^p < 0.05, compared with the elevated-type group. *p* < 0.05, statistically significant difference and *p* < 0.01, highly statistically significant difference indicates in bold.

### 3.4 Radiographic outcomes

**Figure 5:**
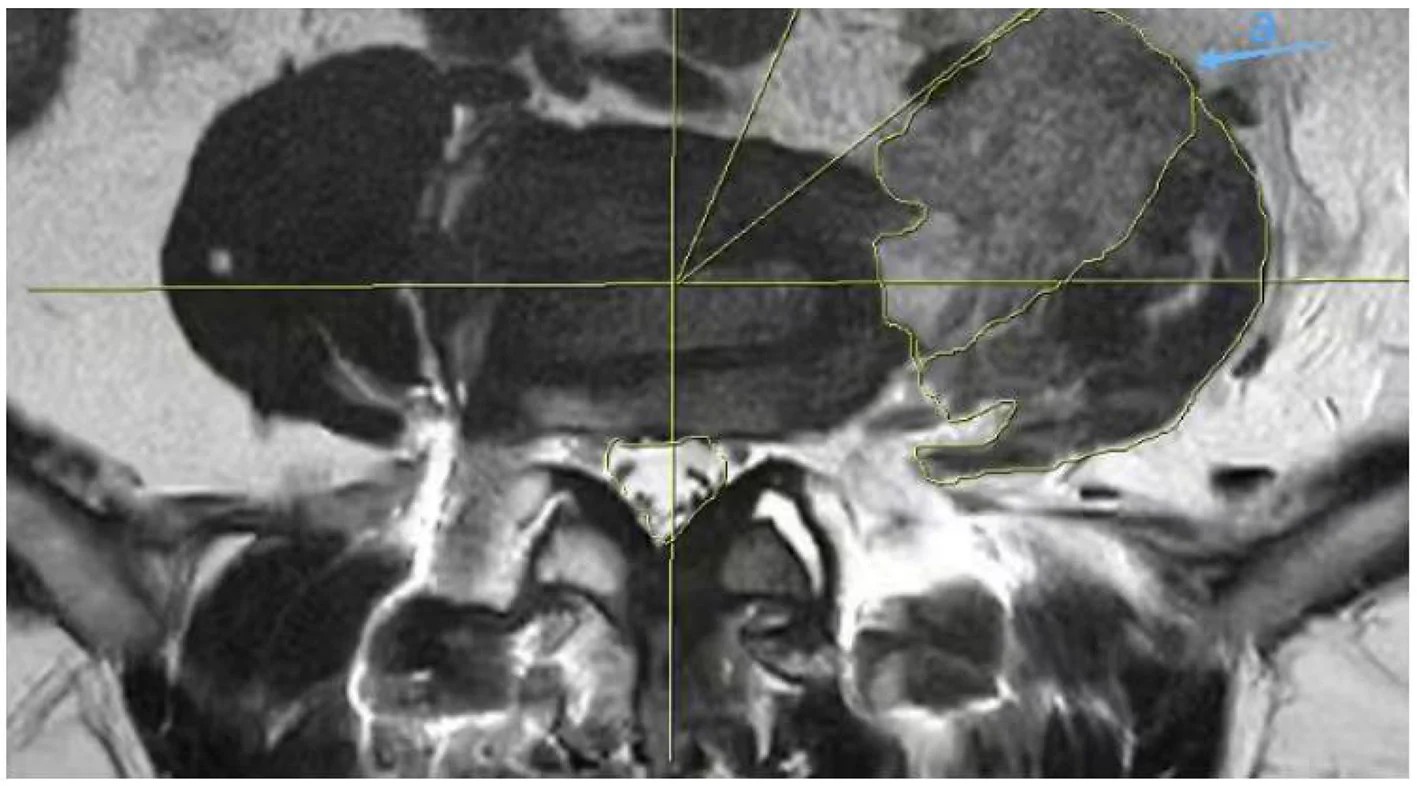
Post-operative psoas major hematoma. Major hematoma area **(a)**.

**Figure 6:**
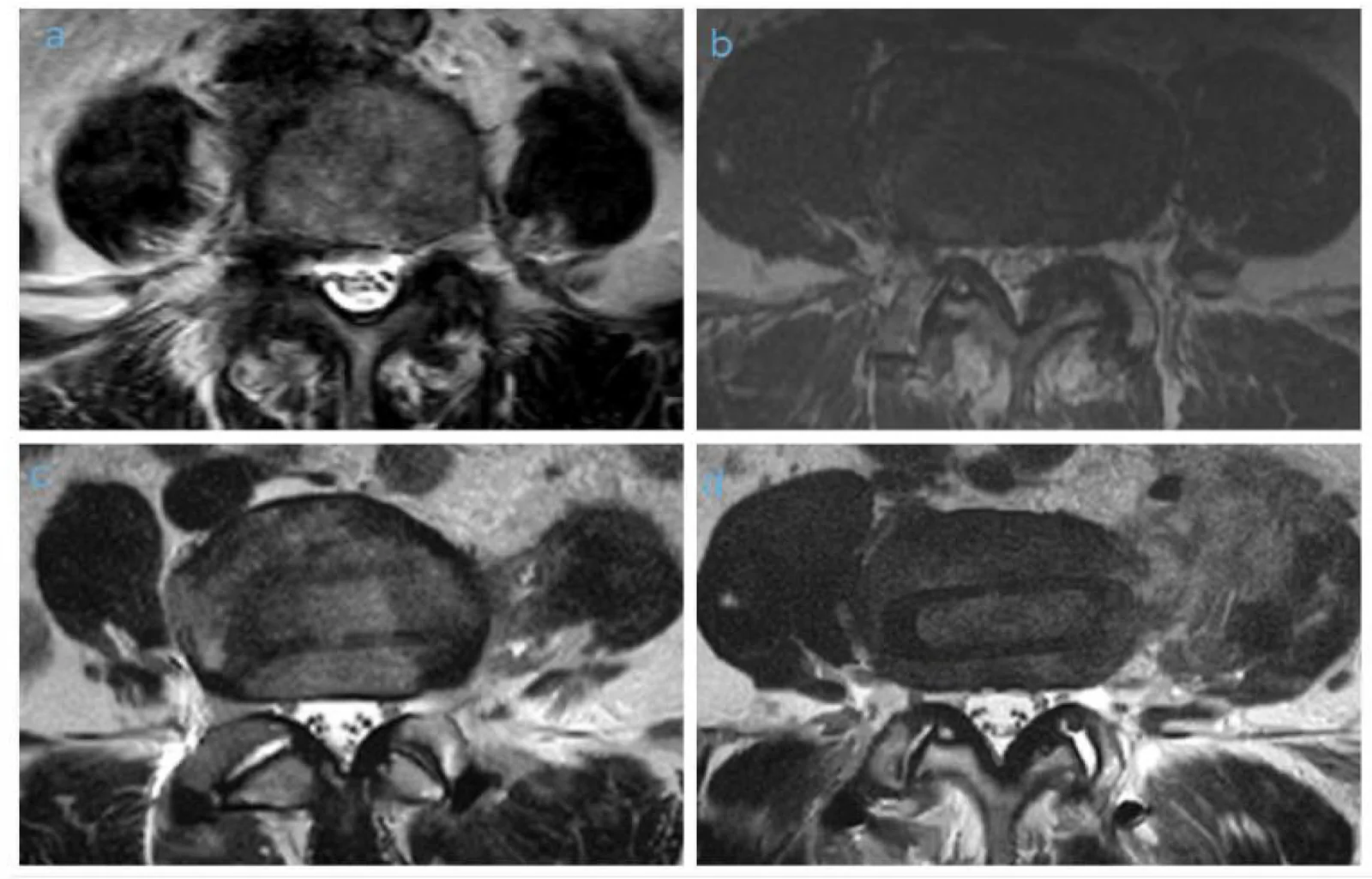
Comparison between elevated type and apposed type psoas major pre- and post-operatively. Pre-operative elevated type psoas major **(a)**, pre-operative apposed type psoas major psoas major **(b)**, post-operative elevated type psoas major **(c)**, post-operative apposed type psoas major **(d)**.

Regarding radiographic parameters, the pre-operative PMCSA at the L4-5 level was 1013.92 ± 269.41 mm^2^ in the elevated-type group and 1046.59 ± 187.40 mm^2^ in the apposed-type group, with no statistically significant difference between the groups. Post-operatively, the PMCSA in the apposed-type group was significantly greater than that in the elevated-type group (1448.52 ± 269.97 mm^2^ vs. 1242.42 ± 331.01 mm^2^, *p* = 0.007). The post-operative PMHA was 306.78 ± 27.69 mm^2^ in the elevated-type group and 486.79 ± 46.78 mm^2^ in the apposed-type group, with a significantly larger hematoma area observed in the apposed-type group (*p* = 0.027). Similarly, the psoas swelling percentage was significantly higher in the opposed-type group than in the elevated-type group (38.42 ± 7.11% vs. 14.38 ± 4.70%, *p* =0.005) (Figures 5, 6). The pre-operative OW was 17.45 ± 3.01 mm in the elevated-type group and 15.81 ± 3.74 mm in the apposed-type group. The OW was significantly narrower in the apposed-type group (*p* < 0.01). Regarding PMSD, PMTD, ADH, PDH, CSCA, and SLA, no statistically significant differences were observed between the two groups (Table 4).

**Table 4:** Imaging parameters of the elevated-type and the apposed-type.

Parameter	Elevated-type		Apposed-type		*p*-value
	Pre-op	3-days post-op	Pre-op	3-days post-op	
OW	17.45 ± 3.01	17.36 ± 3.00	15.81 ± 3.74^*^	15.52 ± 3.69	**< 0.001**
IE	5.41 ± 0.87	5.03 ± 1.71	1.89 ± 0.37^#^	2.41 ± 0.69	**< 0.001**
PMSD	17.51 ± 3.34	20.22 ± 3.90	23.17 ± 3.92	29.64 ± 4.79	0.496
PMTD	29.42 ± 6.30	31.71 ± 5.09	29.56 ± 6.11	35.42 ± 6.10	0.547
PMCSA	1013.92 ± 269.41	1242.42 ± 331.01	1046.59 ± 187.40	1448.52 ± 269.97^₠^	**0.007**
PMHA		306.78 ± 27.69		486.79 ± 46.78^₣^	**0.027**
Swelling in percent		14.38 ± 4.70		38.42 ± 7.11^ⅎ^	**0.005**
CSCA	61.74 ± 21.70	158.60 ± 27.61	57.68 ± 26.84	163.20 ± 30.21	0.746
ADH	12.05 ± 3.74	21.89 ± 2.12	13.50 ± 2.66	20.87 ± 2.47	0.640
PDH	11.02 ± 2.78	20.61 ± 2.48	11.69 ± 1.87	20.20 ± 1.61	0.117
L4-5 SLA	13.25 ± 2.48	14.10 ± 1.74	12.61 ± 2.64	14.36 ± 2.30	0.271

OW, operative window; IE, interior elevation; PMSD, psoas major sagittal diameter; PMTD, psoas major transverse diameter; PMCSA, psoas major cross-sectional area; PMHA, psoas major hematoma area; CSCA, central spinal canal area; ADH, anterior disc height; PDH, posterior disc height; SLA, segmental lordotic angle; ^*^p < 0.01, compared with the elevated-type pre-operation group; ^#^*p* < 0.01, compared with the elevated-type pre-operation group; ^₣^*p* < 0.01, compared with the elevated-type 3-days post-operation group; ^₠^*p* < 0.05, compared with the elevated-type 3-days post-operation group; ^ⅎ^*p* < 0.01, compared with the elevated-type 3-days post-operation group. *p* < 0.05, statistically significant difference and *p* < 0.01, highly statistically significant difference indicates in bold.

### 3.5 Complications

Regarding post-operative complications, 24 patients (24/115, 20.80%) in the apposed-type group experienced complications, compared with seven patients (7/76, 9.50%) in the elevated-type group (*p* = 0.012). The overall complication rate was significantly higher in the apposed-type group (*p* = 0.012). The most common complications included anterior thigh numbness, pain, and hip flexion weakness. One patient in the elevated-type group developed a post-operative decrease in lower limb skin temperature to 28 °C, with a bilateral temperature difference exceeding 1 °C. Additionally, the plantar skin was dry and lacked sweating function.

### 3.6 Regression analysis

Regression analysis was performed to evaluate the associations among pre-operative OW, IE, post-operative psoas swelling percentage, and post-operative complications. Since OW, IE, and percentage swelling show correlation with the incidence of post-operative complications, these three variables were included in the correlation regression analysis. Pre-operative OW was negatively associated with the incidence of post-operative complications (*p* < 0.01). Similarly, pre-operative IE demonstrated a significant negative correlation with post-operative complications (*p* < 0.01). There was no significant association between post-operative psoas swelling percentage and the occurrence of complications (Table 5).

**Table 5:** Univariate regression analysis of multiple variables of risk factors (*n* = 191).

Independent variable	OR	95% CI	*p*-value
OW	0.641	0.835–1.778	**0.004**
IE	0.882	1.069–1.366	**< 0.001**
Swelling in percent	2.857	0.780–10.469	0.827

CI, confidence interval; OR, odds rate; OW, operative window; IE, interior elevation. *p* < 0.05, statistically significant difference and *p* < 0.01, highly statistically significant difference indicates in bold.

## 4 Discussion

OLIF alleviates neurological symptoms by restoring intervertebral disc height, enlarging the intervertebral foramen, and reducing hypertrophy of the ligamentum flavum, thereby relieving neural compression caused by disc space collapse, foraminal stenosis, and ligamentum flavum thickening (21–23). In addition, the use of larger interbody cages combined with posterior fixation effectively mitigates low back pain associated with mechanical instability (4, 24). However, the morphology of the PM—particularly its appositional relationship with the vertebral body at the L4-5 level—may substantially influence intraoperative exposure, the establishment of the surgical corridor, and the operative manipulation. Our findings demonstrated that the apposed-type PM was associated with greater intraoperative blood loss, a narrower operative corridor, more pronounced psoas muscle swelling, and a higher incidence of transient post-operative complications. Moreover, patients in the apposed-type group exhibited worse early post-operative outcomes, as reflected by higher VASB and VASL at 1 week post-operatively.

Although OLIF avoids disruption of posterior spinal elements, it inevitably requires mobilization of the abdominal aorta, hypogastric plexus, and lumbar plexus, thereby increasing the risk of injury to retroperitoneal vessels, muscles, and neural structures. These factors may contribute to symptoms associated with injury to the sympathetic chain and the iliopsoas muscle (12). Previous studies have reported that the incidence of sensory nerve injury and psoas weakness following OLIF ranges from 11.4% to 48.5% (10, 25–27). In the present study, post-operative complications occurred in 31 patients, among whom 30 (30/191, 15.71%) presented with anterior thigh pain or numbness and hip flexion weakness, consistent with previously reported rates. These symptoms are primarily attributed to injury of the lumbar plexus within or adjacent to the PM during retraction. The lumbar plexus, formed by the anterior rami of the T12 and L1–L4 spinal nerves, is embedded within the posterior portion of the PM and extends along the posterior abdominal wall into the lower extremity (28). It consists of six major branches, among which injury to the femoral or obturator nerve may result in sensory deficits in the anteromedial thigh and weakness in hip flexion, knee extension, and thigh adduction (29).

This anatomical relationship implies that any surgical approach traversing or retracting the PM—particularly OLIF—places the lumbar plexus at risk of direct or indirect injury, including compression, traction, and compromised vascular perfusion. Among iatrogenic lumbar plexus injuries, the L4-5 level has been reported to have the highest incidence of post-operative complications, ranging from 3.7% to 66.7% (30). In contrast to OLIF, lateral lumbar interbody fusion (LLIF) involves direct splitting of the PM, which is associated with a higher incidence of neuromuscular complications (31). Given that the lumbar plexus is embedded within the PM, it is particularly susceptible to mechanical forces; transient high-intensity compression may result in more severe injury than prolonged low-intensity pressure (17). The most vulnerable structures are branches of the lumbar plexus, particularly the femoral nerve. Additionally, the genitofemoral nerve, coursing along the psoas surface, is susceptible to injury, potentially leading to sensory disturbances in the groin and anterior thigh. Although intraoperative neuromonitoring (IONM) is considered a standard and essential tool to mitigate neural injury (32), complications remain relatively common.

In this study, one case of lumbar sympathetic chain injury (LSCI) was identified. LSCI is a recognized but often underreported complication of OLIF, with reported incidence rates ranging from 0.5% to 7.3%, likely due to the lack of standardized diagnostic criteria (33). The mechanism is typically iatrogenic, involving compression or traction of the sympathetic chain during surgical manipulation. Clinical manifestations result from temporary or permanent disruption of sympathetic function and include increased skin temperature, anhidrosis, swelling, changes in skin color, and sensory disturbances in the affected limb. These findings were consistent with the clinical presentation observed in our case. This observation suggests that, although OLIF is designed to minimize neural injury, excessive or prolonged tissue retraction may offset these advantages and lead to complications. Accordingly, minimizing both the duration and magnitude of retraction may be critical for preserving neural integrity. Notably, no significant difference in LSCI incidence was observed between the two groups in this study, which may be attributable to under recognition of symptoms or to the relatively small sample size (30).

Wang et al. (18) analyzed 300 patients and introduced the Moro classification, reporting that patients with longer PMSD and narrower OW had poorer short-term post-operative outcomes, higher estimated blood loss, and increased complication rates. Furthermore, IE was negatively associated with complications such as hip flexion weakness and thigh sensory disturbances, whereas a higher psoas swelling percentage was positively associated with these complications. These results suggest that a tighter appositional relationship between the PM and the vertebral body increases the difficulty of surgical exposure, leading to greater compression and prolonged retraction of both the psoas muscle and the lumbar plexus, thereby elevating the risk of post-operative hip flexion weakness and sensory deficits (34).

Regarding clinical outcomes, all patients demonstrated significant improvement in outcome scores during follow-up. Notably, at 1 week post-operatively, VAS scores for both back and leg pain were significantly higher in the apposed-type group than in the elevated-type group (*p* < 0.01). This difference may be attributed to intraoperative psoas compression injury, as hematoma resorption typically requires approximately 2 weeks (16). Most post-operative pain and sensory disturbances generally resolved within 1–3 months. Patients in the apposed-type group exhibited a slightly shorter length of hospital stay compared with those in the elevated-type group. This phenomenon may be attributed to the younger mean age of patients in the apposed-type group; variations in age and BMD could confound hospital stay duration, rather than physiological factors.

Previous studies have described morphological variations of the PM, including a subtype characterized by a longer PMSD and a shorter PMTD at the L4-5 level, commonly referred to as the “teardrop-shaped” psoas (16). Voyadzis et al. (35) suggested that patients with a teardrop-shaped PM may not be suitable candidates for extreme lateral interbody fusion (XLIF), as this configuration may increase the risk of neural injury. In the present study, similar morphological features of the PM were also observed. However, our analysis primarily focused on the appositional relationship between the PM and the vertebral body on the axial plane at the L4-5 level. We demonstrated that tighter apposition between the PM and the vertebral body requires greater retraction force, longer retraction time, and a longer time to establish the surgical corridor. Under such conditions, careful intraoperative identification of the lumbar sympathetic chain becomes particularly important. Moreover, these findings may have significant implications for predicting and reducing post-operative complications.

Hamide et al. (32) reported in their review that the anterior psoas corridor utilized in OLIF reduces the risk of neural injury but at the expense of closer proximity to major vascular structures. In our view, compared with lateral approaches such as LLIF and XLIF, OLIF may reduce the risk of lumbar plexus injury; however, the risk of psoas- and plexus-related complications remains. These inherent trade-offs underscore the importance of individualized surgical planning based on patient-specific anatomical characteristics.

Early gait training consisted of slow, short-step level walking without lumbar torsion, lateral flexion, or excessive forward bending. Walking was performed 4 to 6 times daily, with each session limited to 5 min, and terminated in the absence of aggravated low back pain, leg pain, or numbness. Prolonged sitting, standing, and rapid walking were prohibited during this period. Targeted core stabilization and hip muscle strengthening exercises were performed to improve muscular weakness induced by intraoperative psoas major retraction and enhance walking stability. Straight-line walking and weight-shift training were implemented, and lumbar brace protection was maintained during daily activities. The lumbar brace was removed, and full-weight-bearing independent walking was resumed. Long-distance level walking and slow stair climbing training were performed following the principle of “unaffected limb first when ascending and affected limb first when descending” (36, 37).

This study has several limitations. First, as a retrospective study, it is subject to potential selection bias. Future multicenter studies with larger sample sizes and prospective designs are warranted. Second, the follow-up duration was relatively short, precluding assessment of the long-term impact of psoas morphology on OLIF outcomes. An extended follow-up is needed to evaluate mid- to long-term clinical efficacy. Finally, this study was limited to the L4-5 level, and it remains unclear whether similar effects exist in multilevel procedures. Future studies incorporating subgroup analyses (e.g., two- or three-level surgeries) are needed to elucidate the influence of psoas morphology on OLIF outcomes.

## 5 Conclusions

The appositional relationship between the PM and the vertebral body at L4-5 significantly influences short-term post-operative results. A tightly apposed PM, characterized by a smaller IE and a narrower OW, is associated with inferior early clinical outcomes. Furthermore, this configuration is linked to increased intraoperative blood loss, greater post-operative psoas swelling, and a higher incidence of post-operative complications.

## Data Availability

The raw data supporting the conclusions of this article will be made available by the authors, without undue reservation.
